# Kombucha polysaccharide alleviates DSS-induced colitis in mice by modulating the gut microbiota and remodeling metabolism pathways

**DOI:** 10.3389/frmbi.2024.1341824

**Published:** 2024-02-07

**Authors:** Zhong-Hao Ji, Wen-Yin Xie, Pei-Sen Zhao, Wen-Zhi Ren, Hong-Juan Jin, Bao Yuan

**Affiliations:** ^1^ Department of Laboratory Animals, College of Animal Sciences, Jilin University, Changchun, Jilin, China; ^2^ Department of Basic Medicine, Changzhi Medical College, Changzhi, Shanxi, China; ^3^ Department of Plastic Surgery, The First Hospital of Jilin University, Changchun, Jilin, China

**Keywords:** ulcerative colitis, kombucha polysaccharide, gut microbiota, intestinal barrier, tight junction protein

## Abstract

Ulcerative colitis (UC) is incurable, and rational dietary interventions are important in preventing UC. Kombucha is a fermented beverage that originated in China and has a variety of activities, including antioxidant, antibacterial and hypoglycemic activities. The aim of this study was to analyze the protective effect of Kombucha polysaccharide (KP) against UC and determine its mechanism of action. The results showed that KP intervention was effective in alleviating dextran sulfate sodium salt (DSS)-induced colitis symptoms and inhibiting DSS-induced inflammation and oxidative stress. Moreover, KP was able to reduce intestinal permeability, promote the expression of tight junction proteins, and help maintain thrush cell numbers and promote mucus secretion. The 16S rDNA results indicated that KP intervention increased the abundance of *Rikenellaceae_RC9_gut_group* and *Clostridiales_unclassified*. Untargeted metabolomics techniques revealed that KP can reverse DSS-induced disorders in intestinal metabolism. This study demonstrated that KP alleviated DSS-induced colitis by helping maintain intestinal barrier integrity, regulating the gut microbiota and remodeling metabolism pathways. The findings provide a theoretical basis for the application of KP as a dietary supplement for the prevention of UC.

## 1 Introduction

Ulcerative colitis (UC) is a chronic inflammatory disease that severely affects the lives of patients. Its incidence is increasing every year, and there were 50,000 cases of UC in 2023 worldwide (Le Berre et al., 2023). The pathogenesis of UC is unclear, with genetics, the environment, the intestinal barrier, and immunity being the main factors that influence the development of UC (Kobayashi et al., 2020). 5-Aminosalicylic acid, thiopurines, biologics, and small molecule drugs have played a significant role in UC remission and maintenance therapy for UC, but rectal resection is still needed in 10-20% of drug-tolerant patients (Bots et al., 2018; Le Berre et al., 2020; Le Berre et al., 2023). Therefore, it is important to develop a low-cost, highly active, and safe dietary supplement for UC prevention.

Polysaccharides, as a kind of biomolecules, are widely found in animals, plants and microorganisms (Yu et al., 2018). Polysaccharides possess various physiological activities such as antioxidant, antitumor, antiviral, and anti-inflammatory (Tang and Huang, 2018; Guo et al., 2021; Cor Andrejc et al., 2022). Polysaccharides have shown great potential in the prevention and treatment of UC.Lycium barbarum polysaccharide has been shown to alleviate DSS-induced UC by repairing the intestinal barrier and modulating the gut microbiota (Li et al., 2023). Atractylodes macrocephala-derived polysaccharide was able to alleviate DSS-induced UC by modulating the ratio of Th17/Treg cells (Yang et al., 2022). Dandelion polysaccharide was able to significantly suppress DSS-induced inflammation by inhibiting the NF-κB/NLRP3 pathway and activating Nrf2 signaling (Wang et al., 2023).

Kombucha is a fermented beverage that originated in China and is traditionally made using black tea and cane sugar as a base, which is fermented in the presence of symbiotic culture of bacteria and yeast (SCOBY) (Emiljanowicz and Malinowska-Panczyk, 2020; Antolak et al., 2021; Abaci et al., 2022). The addition of a wide range of nonclassical matrices, such as fruits, vegetables, plants and herbs, makes the variety of kombucha even richer (Barakat et al., 2022; Anantachoke et al., 2023; Wu et al., 2023). Kombucha is rich in tea polyphenols and organic acids, which have a variety of activities, such as antioxidant (Jakubczyk et al., 2020), liver protection (Moreira et al., 2022), antimicrobial (Nyiew et al., 2022; Vukic et al., 2023) and hypoglycemic (Mendelson et al., 2023) activities. A polysaccharide extract of novel shiitake mushroom kombucha was shown to have immunomodulatory effects (Sknepnek et al., 2021). Several studies have shown that kombucha exerts an antimicrobial effect by regulating the body’s gut microbiota (Wang et al., 2021; Permatasari et al., 2022; Xu et al., 2022). However, it is still unknown whether polysaccharides from kombucha can prevent colitis.

Therefore, in this study, based on a DSS-induced acute mouse model of colitis, we analyzed the effect of prophylactic supplementation with KP on alleviating UC. The effects of KP on the intestinal flora and metabolism were analyzed by 16S rDNA sequencing and metabolomics techniques. The results of this study will provide a theoretical basis for the use of KP as a dietary supplement for the prevention of UC.

## 2 Materials and methods

### 2.1 Materials

Dextran sulfate sodium salt (DSS) (molecular weight 36-50 kDa) (MP Biomedicals, CA, USA) and Kombucha polysaccharide (KP) were purchased from HANSUYUAN Biology (Hanzhong, China). Enzyme-linked immunoassay (ELISA) kits for IL-1β, IL-6, TNF-α, superoxide dismutase (SOD), malondialdehyde (MDA), total antioxidant capacity (T-AOC), myeloperoxidase (MPO), and lipopolysaccharide (LPS) were purchased from Shanghai Preferred Biotechnology Co. Streptomycin, ampicillin, gentamicin and vancomycin were purchased from Dalian Meilun Biotechnology Co. The PVDF membrane was purchased from Merck Millipore (Billerica, MA, USA), while the other reagents used in western blotting were obtained from Epizyme (Shanghai, China).

The following antibodies were used in this study:

Anti-Muc2 antibody, anti-Claudin 1 antibody, anti-ZO-1 antibody, and anti-Occludin antibody were purchased from Affinity Biosciences (Cincinnati, OH, USA); anti-GAPDH antibody, and anti-rabbit IgG were purchased from Cell Signaling Technology (Danvers, MA, USA).

### 2.2 Animals and experimental design

Forty-eight SPF-grade 6-week-old male BALB/c mice were purchased from Liaoning Changsheng Biotechnology Co. and housed in the barrier facility at Jilin University Laboratory Animal Center. The experimental protocol was approved and licensed by the Animal Ethics and Welfare Committee of Jilin University (SY202305008).

**Figure 1:**
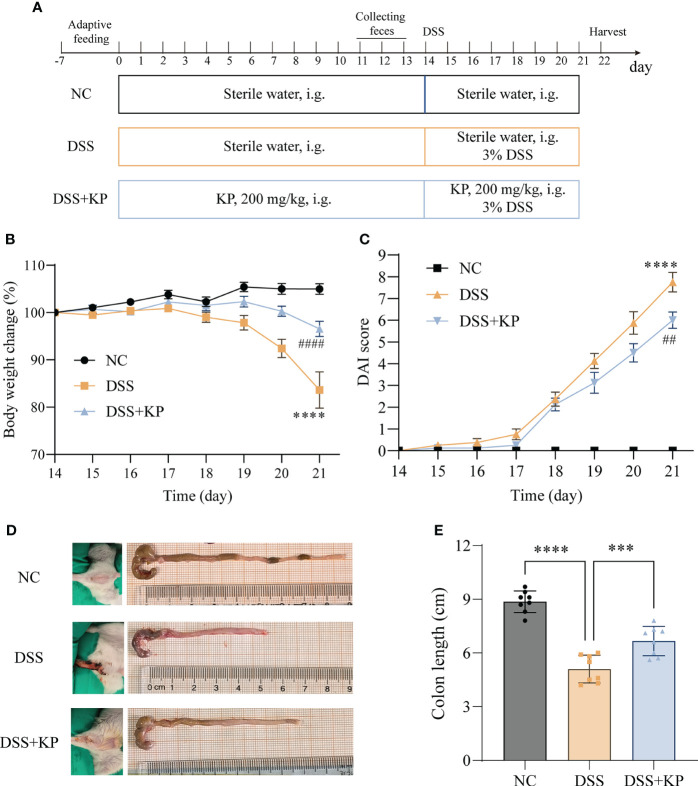
KP relieves DSS-induced colitis symptoms. **(A)** Experimental flow chart; **(B)** weight change curves; **(C)** DAI scores; **(D)** representative images of perianal and colon; and **(E)** colon lengths. (n=8) *** P<0.001, **** P<0.0001; ## P<0.01, #### P<0.0001 (* indicates comparison with the NC group and # indicates comparison with the DSS group in graphs B and C).

After one week of acclimatization, twenty-four mice were randomly divided into three groups, namely, the control group (NC), the model group (DSS) and the KP intervention group (DSS+KP), with 8 mice in each group. The experimental procedure is shown in **Figure 1A**; the NC and DSS groups were gavaged daily with sterile saline, while the treatment group was gavaged daily with 200 mg/kg KP (after referencing to the literature, the three concentrations 50 mg/kg/d, 100 mg/kg/d, and 200 mg/kg/d were utilized in initial tests, and 200 mg/kg/d was finally selected for the formal experiment) (Xiao et al., 2021; Liu et al., 2022; Yang et al., 2023). UC modeling was established by the addition of 3% DSS to the drinking water of the DSS and DSS+KP groups for 7 consecutive days starting on day 14, and the NC group continued to receive distilled water. Disease activity index (DAI) scores were observed and calculated for each mouse daily during the experimental period (Zhang et al., 2022). Observations included percent weight loss, fecal trait score, and fecal blood, and DAI was calculated using the following equation: DAI = (weight loss score + fecal trait score + fecal blood score). Samples of colon, serum and cecum contents were collected on day 22 for subsequent analysis.

### 2.3 Biochemical indicator analysis

The blood samples were collected overnight at 4°C and centrifuged at 3000 rpm for 15 min to collect the supernatant. Colon samples were homogenized using a tissue grinder (1 g of tissue in 9 mL of PBS), and the supernatant was centrifuged at 3000 rpm for 15 min. The levels of IL-1β, IL-6, TNF-α, SOD, MDA and T-AOC in colon tissues and serum were determined, and the levels of MPO and LPS in colon tissues were determined according to the manufacturer’s instructions.

### 2.4 Histopathological staining

Colon tissues were immersed and fixed in 4% paraformaldehyde for 24 h. Paraffin embedding was performed according to the routine procedure, and 5 μm paraffin sections were used for subsequent staining. Hematoxylin-eosin (HE) staining was used to visualize histopathological damage; Alcian blue (AB) and periodic acid-Schiff (PAS) staining were used to observe goblet cells and mucus secretion (Shimizu et al., 2001).

### 2.5 Western blotting and immunohistochemistry

The expression of tight junction proteins (ZO-1, claudin 1, and occludin) in colon tissues was determined by WB. The primary antibodies that were used were an anti-ZO-1 antibody (1:1000), anti-Claudin 1 antibody (1:1000), anti-Occludin antibody (1:1000) and anti-GAPDH antibody (1:2000). The secondary antibody that was used was goat anti-rabbit IgG (1:3000). Tissue proteins were extracted using RIPA. To enhance protein stability, 1% protease inhibitor PMSF was added to RIPA. Protein separation was performed using 10% SDS-PAGE with a protein loading amount of 15 μg. The protein was then transferred to a polyvinylidene-difluoride (PVDF) membrane using the wet transfer method. Subsequently, the membrane was blocked with protein-free rapid blocking solution for 30 minutes at room temperature. After blocking, the membrane was incubated with the primary antibody for 2 hours at room temperature. Following this, the membrane was washed three times with TBST for 10 minutes each. Next, the membrane was incubated with the secondary antibody at room temperature for 1 hour. Post-incubation, the membrane was washed three times with TBST for 10 minutes each. Finally, the blots were visualized with ECL reagent.

IHC was utilized to determine Muc2 expression in colonic tissues. The primary antibodies that were used were anti-Muc2 antibodies (1:200). Detection was performed using a DAB colorimetric kit (Boster, Wuhan, China).

### 2.6 16S rDNA sequencing to analyze the composition of the gut microbiota

The CTAB method was selected for extraction of total DNA from cecum contents. After passing the quality test, the V3+V4 region was amplified using PCR primers (PCR primer sequences: 341F, 5’-CCTACGGGGNGGCWGCAG-3’; and 805R, 5’-GACTACHVGGGTATCTAATCC-3’). PCR amplification products were detected by 2% agarose gel electrophoresis and recovered and purified. Subsequently, 2×250 bp bipartite sequencing was performed using a NovaSeq 6000 sequencer. Bioinformatic analysis was performed using the OmicStudio tools at https://www.omicstudio.cn/. The 16S rRNA sequencing data were analyzed for significant differences using the Kruskal-Wallis test and Dunn’s *post-hoc* test, and q-values were obtained by correcting for multiple comparisons with the Benjamini-Hochberg test (pVal and qVal count tool was performed using the OmicStudio tools at https://www.omicstudio.cn/tool). Gut microbiota beta diversity was analyzed using principal coordinate analysis (PCoA) based on bray_curtis, jaccard and unweighted unifrac distance matrices. Discrete analyses were performed using Analysis of similarities (Anosim) to obtain R-values and P-values. Where the R-value is closer to 1, indicating that the greater the difference between samples between groups, while the smaller the difference between samples within groups, the better the grouping effect, and the P-value reflects the statistical significance of the results of Anosim’s analysis, with P<0.05 indicating whether or not the statistic is significant. Linear discriminant analysis Effect Size (LEfSe) was used to identify differentially abundant taxa with a LDA score >4.0.

### 2.7 Analysis of metabolites by untargeted metabolomics

Metabolites were extracted from the contents of the cecum using 80% methanol, and 0.5 mL of 80% methanol was added to each 50 mg sample and left at -20°C for 30 min, followed by centrifugation at 20,000 × g for 15 min. The supernatant was transferred to a new centrifuge tube and lyophilized. Liquid chromatography tandem mass spectrometry (LC-MS) analysis was performed after reintroducing 100 μL of 80% methanol to dissolve the samples. Samples were scanned separately in positive and negative ion mode by mass spectrometry. Differentially abundant metabolites between groups were analyzed using the wilcox.test, and the Benjamini-Hochberg test was used to correct for multiple comparisons with FDR to obtain the q-value. Supervised partial least squares discriminant analysis (PLS-DA) was performed using metaX, and variable importance for projection (VIP) values were calculated. Thresholds for defining differentially abundant metabolites between groups were multiplicity of differences ≥1.5 or ≤1/1.5, P value <0.05, and VIP value ≥1. Spearman correlation heatmap with signs was performed using the OmicStudio tools at https://www.omicstudio.cn. (no multiplicity correction done).

### 2.8 Statistical analysis

The experimental data are presented as the mean ± standard deviation (SD). One-way ANOVA followed by Dunnett *post-hoc* test was used to compare multiple groups. The data were analyzed and plotted using GraphPad Prism9.5 (La Jolla, CA, USA). All experiments contained at least 3 biological replicates, and P < 0.05 indicated that the differences were statistically significant.

## 3 Results

### 3.1 KP relieves DSS-induced colitis-like symptoms

The effect of dietary supplementation with KP on preventing colitis was evaluated based on a DSS-induced acute colitis mouse model. The results of animal experiments showed that, compared with the NC group, the mice with DSS induction showed weight loss (**Figure 1B**), DAI score elevation (**Figure 1C**), perianal hemorrhage, and colon shortening (**Figures 1D, E**). In contrast, KP intervention was able to significantly alleviate the above symptoms induced by DSS.

### 3.2 KP inhibits DSS-induced inflammation and oxidative stress

**Figure 2:**
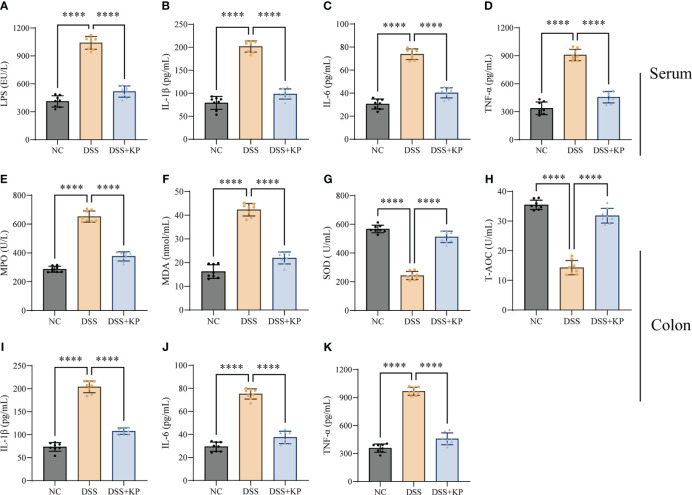
KP inhibits DSS-induced inflammation and oxidative stress. **(A–D)** Serum levels of LPS, IL-1β, IL-6, and TNF-α; **(E–H)** levels of MPO, MDA, SOD, and T-AOC in colonic tissues; and **(I–K)** levels of IL-1β, IL-6, and TNF-α in colonic tissues. (n=8) **** P<0.0001.

Exacerbation of the proinflammatory response and oxidative stress are typical features of colitis (Recinella et al., 2022; Zhang et al., 2022). The effect of KP intervention on DSS-induced inflammation and oxidative stress was analyzed by measuring the expression levels of IL-1β, IL-6, TNF-α, MDA, SOD, T-AOC, LPS, and MPO in serum and colon samples. The results showed that the levels of LPS and three proinflammatory factors in serum were significantly increased (P < 0.0001) after DSS treatment (**Figures 2A–D**); the levels of MPO, MDA, and proinflammatory factors expressed in colon tissues were significantly increased (P < 0.0001) (**Figures 2E, F, I–K**), and the levels of the antioxidant factors SOD and T-AOC were significantly decreased (P < 0.0001) (**Figures 2G, H**). KP intervention significantly suppressed DSS-induced inflammation and oxidative stress.

### 3.3 KP alleviates DSS-induced intestinal barrier damage

**Figure 3:**
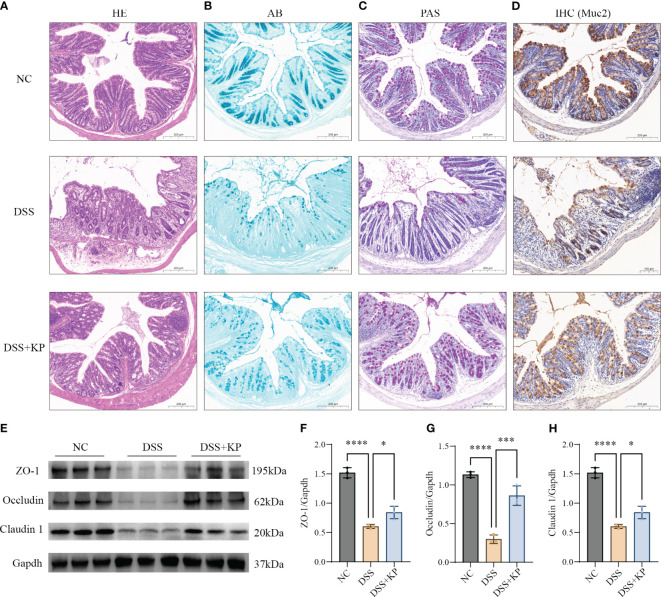
KP alleviates DSS-induced damage to the mechanical intestinal barrier. **(A)** Representative image of HE staining; **(B)** representative image of AB staining, AB-stained positive areas indicate mucus secreted by goblet cells; **(C)** representative image of PAS staining, PAS stains the mucins in the goblet cells a purplish-red color, thus indicating the location of the goblet cells; **(D)** IHC detection of Muc2 protein expression in colonic tissues, Muc2 is a mucin secreted by goblet cells; **(E)** WB detection of protein expression of ZO-1, occludin and claudin 1 in colonic tissues; and **(F–H)** the results of normalization of the ZO-1, occludin and claudin 1 proteins. (n=3) ns P > 0.05, * P < 0.05, *** P < 0.001, **** P < 0.0001.

The physical intestinal barrier is the first barrier that separates the body from harmful substances in the intestines and consists mainly of tight junction proteins, goblet cells and the mucus they secrete (Mehandru and Colombel, 2021; Yao et al., 2022). The effect of KP on intestinal barrier protection was analyzed by pathological staining, IHC and WB. The results showed that DSS induced crypt loss, intestinal wall swelling, inflammatory cell infiltration (**Figure 3A**), mucin reduction, goblet cells loss (**Figures 3B–D**), and a significant decrease in the expression of tight junction proteins (P < 0.0001) (**Figures 3E–H**), whereas KP intervention significantly alleviated DSS-induced intestinal barrier damage and helped maintain intestinal barrier integrity.

### 3.4 KP reversed DSS-induced disorder of the gut microbiota

**Figure 4:**
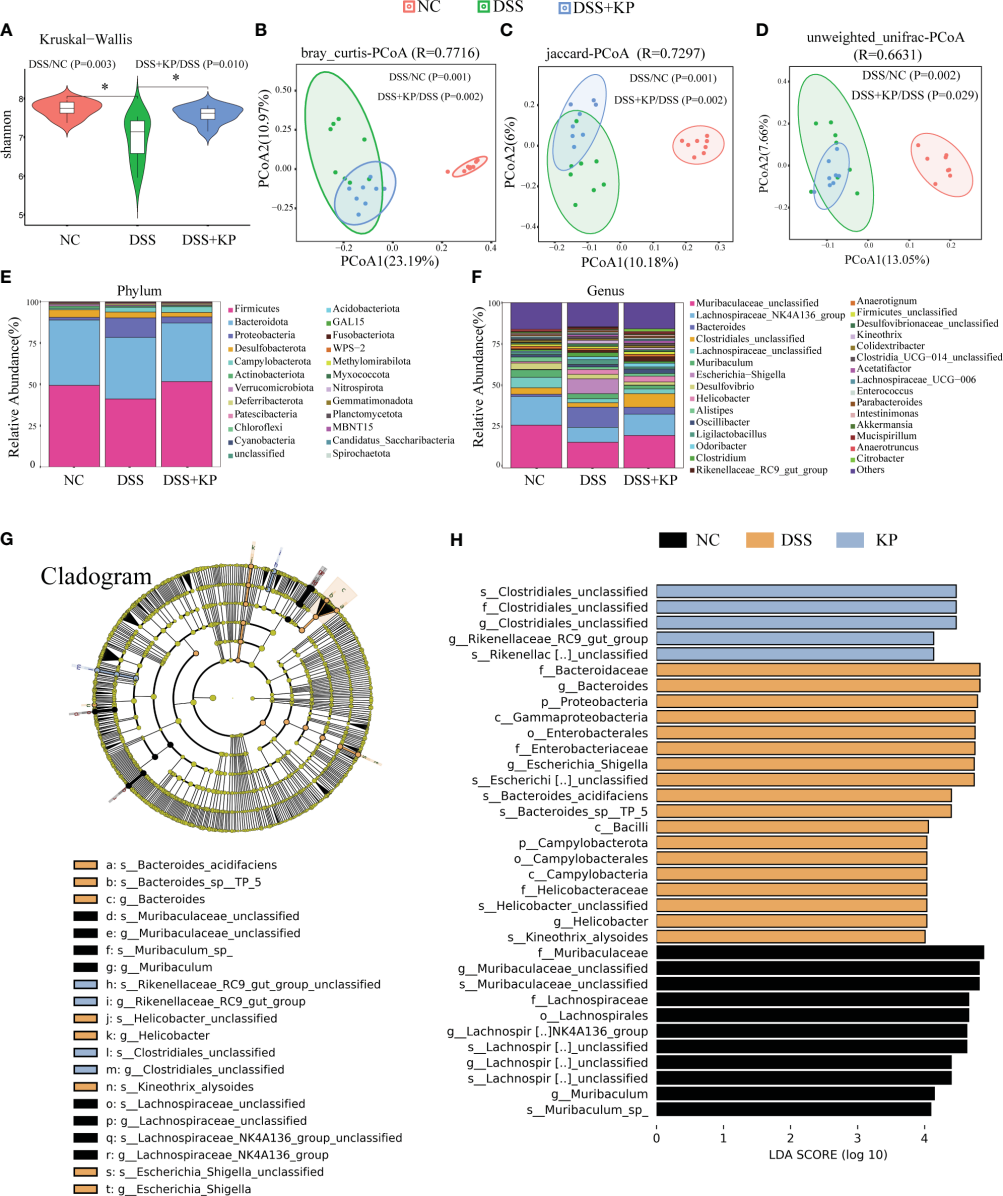
KP alleviates DSS-induced gut microbiota. **(A)** Shannon index; **(B)** PCoA analysis based on bray_curtis distance matrices; **(C)** PCoA analysis based on jaccard distance matrices; **(D)** PCoA analysis based on unweighted unifrac distance matrices; **(E)** microbial composition at the phylum level; **(F)** microbial composition at the genus level; and **(G)** The cladogram of LEfSe analysis; **(H)** LDA scores in differentially abundant taxa (LDA > 4.0). (n=8) * P < 0.05.

The gut microbiota is an ecological barrier that maintains intestinal homeostasis, and altered composition and decreased diversity of the gut flora are characteristic of ulcerative colitis, as well as being important factors that influence disease progression and treatment (Costello et al., 2019; Franzosa et al., 2019). 16S rDNA sequencing was applied to analyze gut microbiota composition. The results of alpha diversity analysis showed a significant decrease (P < 0.05) in the Shannon index after DSS induction and a significant increase (P < 0.05) in the Shannon index in the DSS+KP group compared with that of the DSS group (**Figure 4A**). PCoA analysis based on bray_curtis, jaccard and unweighted unifrac distance matrices., were used to assess the β-diversity of samples, and the results showed that there was a significant separation between the DSS group and the NC group, whereas the microbial composition of the DSS+KP group tended to regress toward that of the NC group (**Figures 4B–D**). **Figures 4E, F** show the gut microbiota composition at the phylum and genus levels, more detailed information is shown in **Supplementary Table 1**. LEfSe analysis revealed 9 and 11 differentially abundant genera and species, respectively (LDA score > 4.0) (**Figures 4G, H**). Among them, *Clostridiales_unclassified* and *Rikenellaceae_RC9_gut_group* were the biomarkers of DSS+KP group. In conclusion, these results indicate that KP intervention might effectively reverse at least some aspects of DSS-induced disorder.

### 3.5 KP alleviates DSS-induced disorder of intestinal metabolism

**Figure 5:**
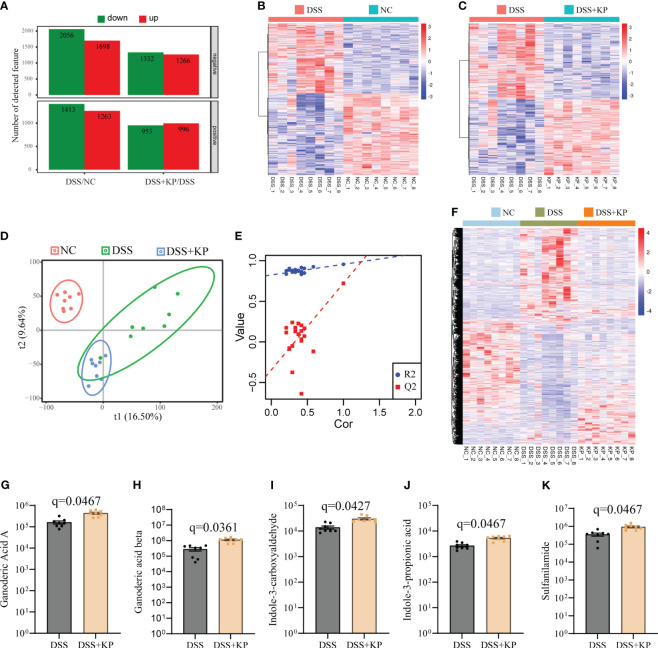
Effect of KP on intestinal metabolism in DSS-induced colitis mice. **(A)** Differentially abundant metabolites between groups in positive and negative ion mode, with the criteria for determining differences as fold change ≥1.5 or ≤1/1.5, P value <0.05, and VIP value ≥1; **(B)** heatmap of differentially abundant metabolites between the DSS group and the NC group; **(C)** heatmap of differentially abundant metabolites between the DSS group and the DSS+KP group; **(D)** PLS-DA score plot; **(E)** PLA-DA analysis of the permutation test results; **(F)** heatmap showing the composition of metabolites in the three groups; and **(G-K)** Ganoderic Acid A, Ganoderic acid beta, Indole-3-carboxyaldehyde, Indole-3-propionic acid and Sulfanilamide 5 metabolites in DSS and DSS+KP groups. (n=8).

LC-MS analysis of metabolites in the cecum contents of the three groups of mice. With a multiplicity of difference ≥1.5 or ≤1/1.5, P <0.05, and a VIP value ≥1 as the defining thresholds, in negative ion mode, the expressions of 2056 metabolites were downregulated and 1698 metabolites were upregulated in the DSS group compared with the NC group; the expressions of 1332 metabolites were downregulated and 1266 metabolites were upregulated in the DSS+KP group compared to the DSS group. In positive ion mode, the expressions of 1413 metabolites were downregulated and 1263 metabolites were upregulated in the DSS group compared with the NC group, and the expressions of 953 metabolites were downregulated and 996 metabolites were upregulated in the DSS+KP group compared with the DSS group (**Figure 5A**). **Figures 5B, C** shows the clustering of differentially abundant metabolites between the two groups in the form of a heatmap. PLS-DA analysis showed that the three groups of mice had different metabolic profiles (**Figures 5D, E**). **Figure 5F** shows the composition of metabolites in the three groups in the form of a heat map and shows that KP intervention can reverse DSS-induced disorder of intestinal metabolism. **Figures 5G–K** specifically shows the abundance of five metabolites (Ganoderic Acid A, Ganoderic acid beta, Indole-3-carboxyaldehyde, Indole-3-propionic acid and Sulfanilamide) in DSS and DSS+KP groups, more detailed information is shown in **Supplementary Table 2**.

**Figure 6:**
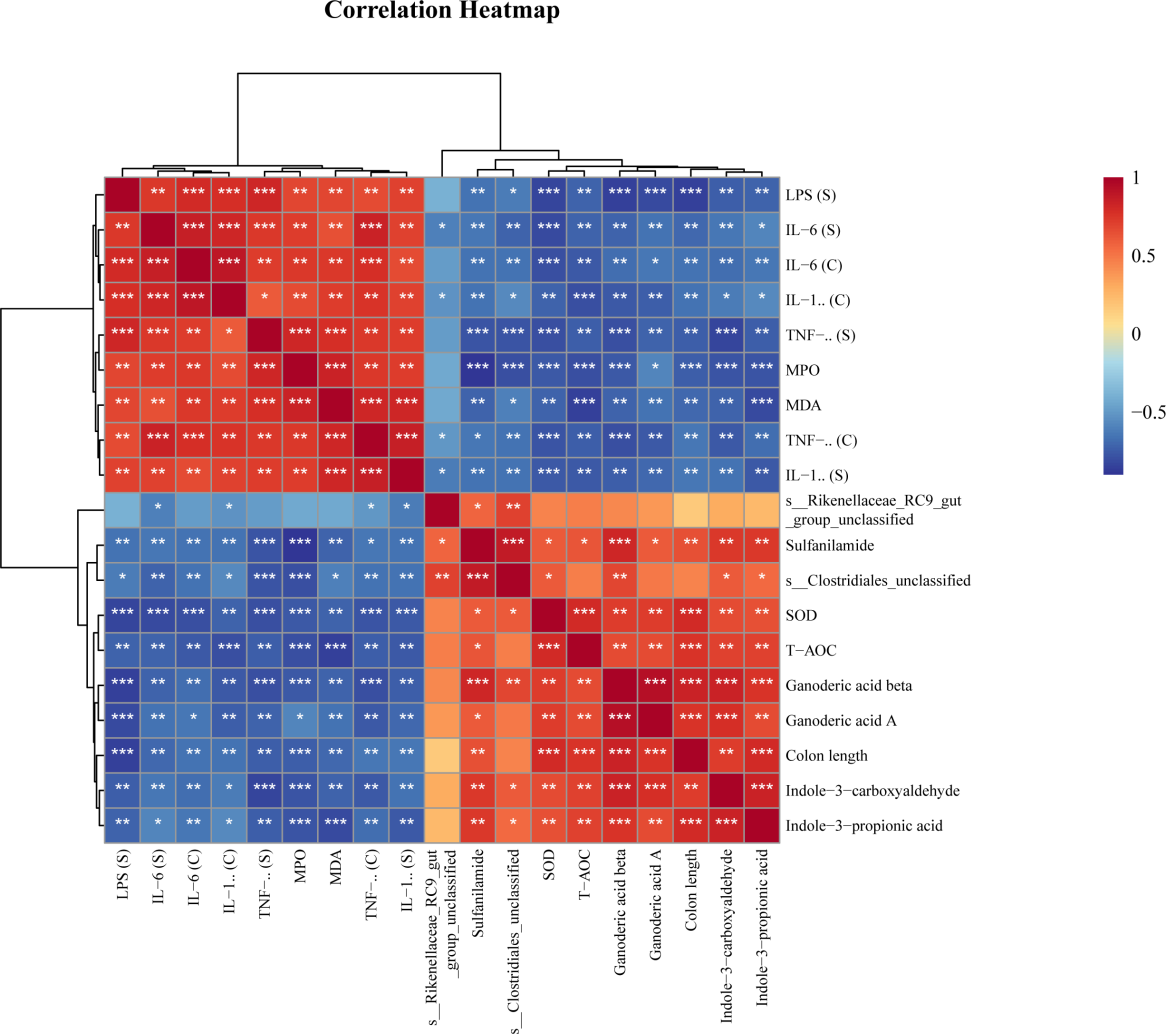
Spearman correlation analysis of key bacteria, metabolites and physiological and biochemical indicators. * P<0.05, ** P<0.01, *** P<0.001.

Spearman analysis was used to reveal correlations between key bacteria, metabolites, and physiological and biochemical indicators. The results showed that *Rikenellaceae_RC9_gut_group* and *Clostridiales_unclassified* were negatively correlated with proinflammatory factors and oxidative indices and positively correlated with antioxidant factors and differentially abundant metabolites (**Figure 6**).

## 4 Discussion

UC is one of the most common inflammatory bowel diseases, with an incidence rate that increases year after year, and its course is characterized by alternating remission-relapse states (Le Berre et al., 2023). There is not yet a cure for UC, and the current goal is to alleviate symptoms and prevent and treat complications (Gros and Kaplan, 2023). The introduction of the food-as-medicine concept has led to the realization that improved health can be achieved through nutrition (Mozaffarian et al., 2022). Numerous studies have shown that natural polysaccharides exhibit great potential in the prevention and treatment of UC by influencing the intestinal microflora (Guo et al., 2021; Xu et al., 2023), regulating metabolism (Liu et al., 2022; Xu et al., 2023), maintaining the integrity of the intestinal barrier (Xiao et al., 2021), and improving immunity (Zou et al., 2023). Kombucha is a fermented beverage that has several beneficial effects in the body, and in this study, based on a DSS-induced acute UC model, we found that dietary supplementation with KP significantly alleviated the symptoms of DSS-induced colitis and suppressed inflammation and oxidative stress.

Impaired intestinal barrier integrity is a typical feature of UC. The intestinal barrier includes the mechanical intestinal barrier, the microbial barrier, and the immune barrier. Our findings demonstrate that dietary supplementation with KP reduces intestinal permeability, promotes the expression of tight junction proteins, helps maintain goblet cells numbers and promoting mucus secretion. Additionally, dietary supplementation with KP increases the diversity of the gut microbiota and modulates the composition of gut microbiota. *Rikenellaceae_RC9_gut_group* produces propionic acid and butyric acid using succinic acid or lactic acid as substrates (Hosomi et al., 2022). Functionally, *Rikenellaceae_RC9_gut_group* regulates lipid metabolism and glucose metabolism homeostasis (Zhou et al., 2018; Sebastia et al., 2023) and alleviates oxidative stress (Tang et al., 2024). In addition, ginsenoside Rg1 was reported to alleviate ulcerative colitis in obese mice, whereas ginsenoside Rg1 was able to up-regulate the abundance of the *Rikenellaceae_RC9_gut_group* in terms of gut microbiota composition (Zhong et al., 2023). The function of *Clostridiales*_unclassified is associated with short-chain fatty acid metabolism (Tsukuda et al., 2021; Tian et al., 2022). The abundance of *Rikenellaceae_RC9_gut_group* and *Clostridiales_unclassified* were differentially abundant after KP intervention. These results suggest that KP can alleviate gut microbiota imbalance and prevent colitis.

Metabolic disorders occur in patients with IBD, and metabolites show promise for the diagnosis, staging, and treatment of IBD (Williams et al., 2009; Martin et al., 2016; Bjerrum et al., 2021). Multiple drugs inhibit intestinal inflammatory responses by remodeling tryptophan metabolism and bile acid metabolism (Liu et al., 2022; Xiao et al., 2022; Wang et al., 2023; Yan et al., 2023). KP intervention was able to significantly increase the abundance of metabolites such as Ganoderic Acid A, Ganoderic acid beta, Indole-3-carboxyaldehyde, Indole-3-propionic acid and Sulfanilamide. Ganoderic Acid A inhibits the NF-κB pathway and the TLR4/NLRP3 pathway (Wang et al., 2022; Zheng et al., 2022). Ganoderic acid A protects against inflammatory diseases by regulating the intestinal flora, and the T helper cell 17/regulatory T-cell balance (Zhang et al., 2021; Lv et al., 2022). In addition, ganoderic acid beta promotes the production of the anti-inflammatory factor IL-10 (Liu et al., 2022). Indole-3-propionic Acid maintains intestinal barrier integrity by promoting tight junction protein expression and mucus secretion (Li et al., 2021). Indole-3-carboxaldehyde activates the Nrf2/HO-1 signaling pathway, inhibits ROS production and exerts antioxidant functions (Lu et al., 2023). Therefore, we hypothesized that KP may inhibit inflammation and oxidative stress and maintain intestinal barrier homeostasis by remodeling metabolism and increasing the levels of beneficial metabolites such as Ganoderic Acid A, Ganoderic acid beta, Indole-3-carboxyaldehyde, and Indole-3-propionic acid.

Using the body surface area method to convert equivalent doses between mice and humans, it is determined that the dose for mice is approximately 12.3 times higher than that for humans. Specifically, the dose administered to mice is 200mg/kg, thus resulting in a dose of 1138mg for a person weighing 70kg. Furthermore, it is worth noting that the aforementioned dose of 200 mg/kg in mice did not elicit any discernible acute toxic side effects. Nevertheless, to comprehensively assess its toxicological and side effects, further investigations involving chronic toxicity experiments are imperative.

In conclusion, in this study, we demonstrated that KP alleviated DSS-induced colitis by helping maintain intestinal barrier integrity, modulating the gut microbiota and remodeling metabolism pathways. The results of the study will provide a theoretical basis for the use of KP as a dietary supplement for the prevention of UC.

## Data availability statement

The data presented in the study are deposited in the National Genomics Data Center repository, accession number CRA013559.

## Ethics statement

The animal study was approved by Animal Ethics and Welfare Committee of Jilin University. The study was conducted in accordance with the local legislation and institutional requirements.

## Author contributions

Z-HJ: Conceptualization, Formal analysis, Methodology, Writing – original draft. W-YX: Data curation, Methodology, Writing – original draft. P-SZ: Data curation, Methodology, Visualization, Writing – review & editing. W-ZR: Supervision, Writing – review & editing. H-JJ: Conceptualization, Funding acquisition, Writing – review & editing. BY: Conceptualization, Funding acquisition, Supervision, Writing – review & editing.

## Supplementary material

Supplementary Table 1 Results of the analysis of differences in microbial composition among the three groups.

Supplementary Table 2 Differential metabolites between DSS and DSS+KP groups (q<0.05).
